# Supplementary material for: Evidence-Based Recommendations for Herd Health Management of Porcine Post-Weaning Diarrhea
Source: Animals (Basel). 2022 Jul 6;12(14):1737. doi: 10.3390/ani12141737 (PMC9311872; doi:10.3390/ani12141737)

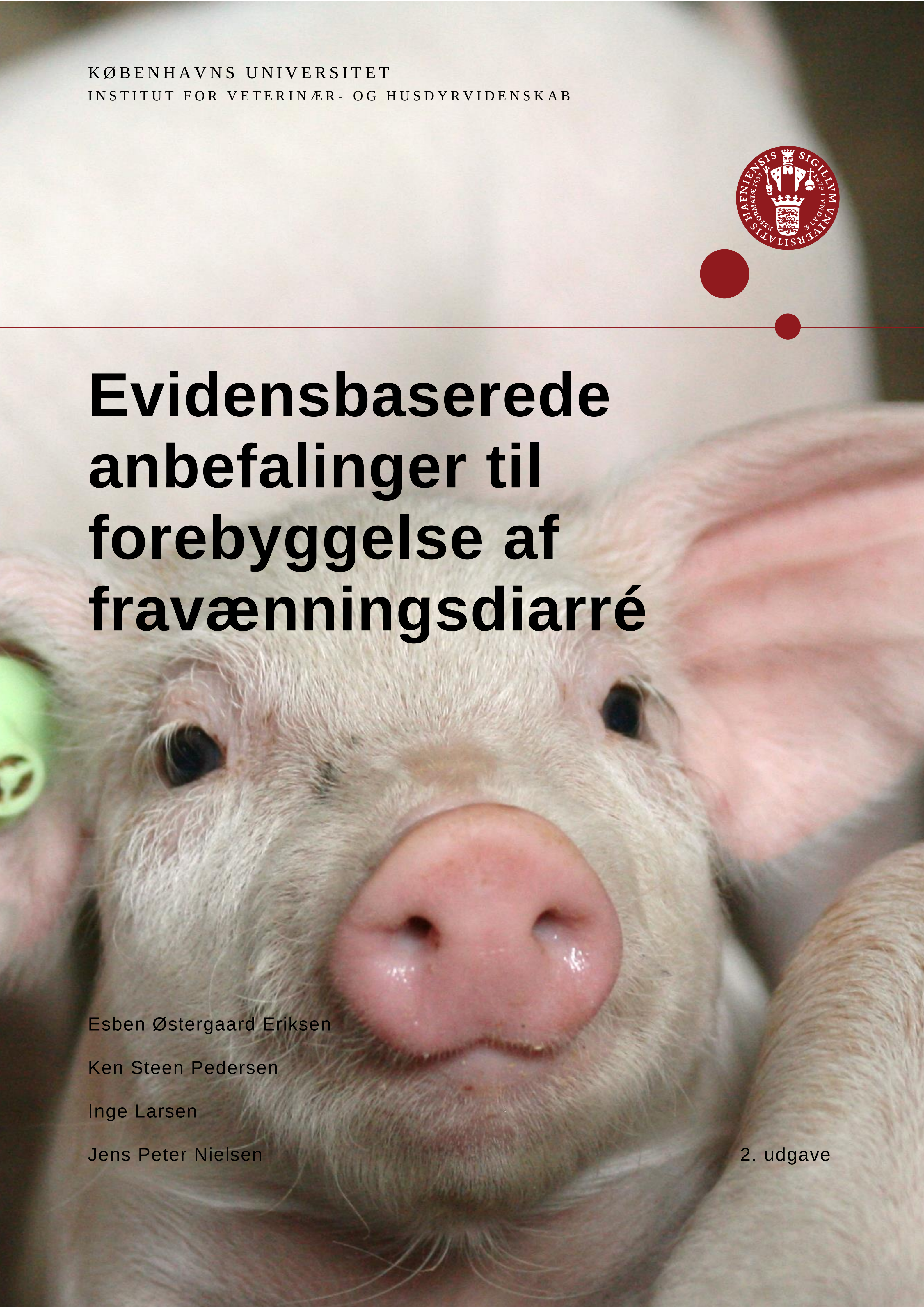

KØBENHAVNS UNIVERSITET
INSTITUT FOR VETERINÆR- OG HUSDYRVIDENSKAB

Evidensbaserede anbefalinger til forebyggelse af fravænningsdiarré

Esben Østergaard Eriksen

Ken Steen Pedersen

Inge Larsen

Jens Peter Nielsen

2. udgave

Evidensbaserede anbefalinger til forebyggelse af fravænningsdiarré

2. udgave, juli 2022

Af Esben Øster Eriksen, Ken Steen Pedersen, Inge Larsen, og Jens Peter Nielsen
Forside- og bagsidebillede: Inge Larsen

Evidensbaserede anbefalinger til forebyggelse af fravænningsdiarré er lavet i løbet af Esben Østergaard Eriksens Ph.d.-studium som en del af forskningsprojektet *Pigs raised without the use of antibiotics (RWA-production)* finansieret af Miljø- og Fødevareministeriet gennem Grønt Udviklings- og Demonstrationsprogram (GUDP). Den 2. udgave er ganske let revideret i forhold til 1. udgave som blev dissemineret til danske svinedyrlæger i marts 2022. Den 2 udgave er publiceret som ekstramateriale til artiklen, hvor det komplette forskningsarbejde er beskrevet på engelsk:
Eriksen, E.Ø.; Pedersen, K.S.; Larsen, I.; Nielsen, J.P.
Evidence-Based Recommendations for Herd Health Management of Porcine Post-Weaning Diarrhea
Animals 2022, 12. Special Issue: Porcine Health Management

Københavns Universitet
Institut for Veterinær og Husdyrvidenskab
Sektion for produktion ernæring og sundhed
Grønnegårdsvej 2
1870 Frederiksberg C

esbene@sund.ku.dk

Indhold

Indhold 2
Introduktion 4
Metode 4
Genetik 5
Avlslinje/race 5
E. coli-resistente dyr 5
Management før fravænning 6
Alder ved fravænning 6
Kropsvægt ved fravænning 6
Råmælksindtag 7
Fødselsvægt 7
Fodring i dieperioden 9
Supplerende fodring med mælkeerstatning i dieperioden 10
Vaccination af søer 10
Antibiotikabehandling i dieperioden 11
Delfravænning af kuld 11
Management under fravænningen 12
Gradvis fravænning 12
Håndtering under fravænning/ flytning af grisene 14
Transport 15
Fravænning i farestien 15
Management efter fravænning 16
Belægningsgrad 16
Gruppestørrelse per sti 16
Sortering af grisene i stierne 17
Rode- og beskæftigelsesmateriale 17
Rumtemperatur og luftflow 18
Luftkvalitet 18
Foder- og vandtildelingsstrategi 19
Plads ved fodertruget 19
Fodringsregime 19
Gulv-/måttefodring 20
Grødfoder 20
Foderets partikelstørrelse 21
Foderets fysiske form 21
Vandforsyning 22
Smittebeskyttelse og smittehåndtering 23
Rengøring før indsættelse 23
Alt ind/ alt ud praksis 23
Personale og deres tøj som potentielle fomitter 24
Arbejdsrutine fra de yngste til de ældste grise 24
Renhold af potentielle fomitter 25
Renhold af transportvogn 25

Rent drikkevand 25
Management af specifikke patogener 26
Vaccination mod ETEC 26
Vaccination mod rotavirus 26
Referencer 27

Introduktion

Fravænningsdiarré er en multifaktoriel tilstand karakteriseret ved det kliniske tegn diarré de første 14 dage efter fravænning. Tilstanden forekommer, når grise fravænnes abrupt og tidligere end tidspunktet for naturlig fravænning, og kan derfor henregnes som en produktionssygdom. Grisen selv, dennes miljø og fodring, samt hvilke patogener den eksponeres for, udgør tilsammen et komplekst årsagsforhold. Det gør det vanskeligt, men også fagligt interessant og udfordrende at finde opskriften til at forebygge fravænningsdiarré i en given besætning. Formålet med nærværende anbefalinger til forebyggelse af fravænningsdiarré er at give et overblik over, hvilke anbefalinger, der understøttes af forskningsbaseret evidens. Med dette overblik bliver det forhåbentlig nemmere at overveje og udvælge tiltag til forebyggelse af fravænningsdiarré uden brug af zink og antibiotika. Anbefalingerne er i sprog og form målrettet dyrlæger, men kan også anvendes af andre rådgivere samt interesserede svineproducenter og staldpersonale.

Alle anbefalingerne egner sig til taktiske beslutninger i den intensive indendørs produktion af grise som den typisk drives i Danmark anno 2022. Ved beslutninger på taktisk niveau skal forstås, at anbefalingerne umiddelbart kan implementeres med ændringer inden for produktionens nuværende form. Det betyder samtidig også, at der ikke lægges op større strategiske beslutninger om svinebedriftens indretning, for eksempel giver vi ikke anbefalinger som ultimativt kræver opførsel af nye staldbygninger eller omlægning af driftsformen. Anbefalingerne begrænser sig også til management-relaterede forhold, herunder hvordan og på hvilken form foder tildeles. Der gives ikke anbefalinger for selve fodersammensætninger eller foder- og vandtilskud, og det vil derfor være relevant at konsultere andre informationskilder eller eksperter for at gennemgå disse vigtige forhold, når risikoen for fravænningsdiarré skal minimeres i en besætning. Det må forventes, at effekten af et givent tiltag ofte vil være besætningsspecifik, og det anbefales derfor at foranstalte egentlige besætningsafprøvninger, hvor der indsamles data for udvalgte effektmål før og efter de anbefalede tiltag iværksættes.

Anbefalingerne er inddelt i temaerne: Genetik, Management før fravænning, Management under fravænningen, Management efter fravænning, Foder- og vandtildelingsstrategi, Smittebeskyttelse og smittehåndtering og Management af specifikke patogener.

Metode

Anbefalingerne er udarbejdet ved følgende fremgangsmåde. I tre databaser søgte vi efter videnskabelige oversigtsartikler (reviews) udgivet efter år 2000, hvor andre forskere havde opsummeret den tilgængelige evidens om temaer relateret til fravænningsdiarré. Vi udarbejdede en liste med punkter relateret til fravænningsdiarré efter samråd med en række danske eksperter, og vi tilføjede ekstra punkter, der blev nævnt i den litteratur vi fandt. For hvert punkt blev den videnskabelige konsensus og konkrete anbefalinger høstet eller syntetiseret fra oversigtsartiklerne. Vi fokuserede først og fremmest på, om der var evidens for punktets effekt på forekomsten af klinisk diarré umiddelbart efter fravænning. Dernæst om der var evidens for punktets effekt på grisenes præstation og produktivitet efter fravænning og punktets effekt på markører for tarmsundhed, fx histologiske, mikrobiologiske eller molekylære undersøgelser af materiale fra tarmen. Hvis vi ved sorteringen af vores søgeresultater ikke fandt nye og gode oversigtsartikler, som afdækkede et givent punkt, foretog vi en ny søgning efter oversigtsartikler for det konkrete punkt, og ultimativt søgte vi efter originale forskningsartikler for selv at lave en opsummering af den tilgængelige evidens. Denne fremgangsmåde afdækker sandsynligvis ikke alt den relevante litteratur. For nogle punkter var der ingen, meget sparsom eller tvivlsom evidens, og her kunne vi således ikke komme med konkrete anbefalinger. Her bør det bemærkes, at et fravær af evidens ikke er det samme som en antydning af, at noget ikke har effekt, blot at vi ikke kan sige noget om det med baggrund i forskningsdata.

Genetik

Avlslinje/race

Hvad ved vi?

Tarmmikrobiotaen er forskellig for grise af forskellige avlslinjer/race, der holdes i det samme miljø. Studier, der har undersøgt sammenhængen mellem avlslinjer og forekomsten af fravænningsdiarré og/eller markører for tarmsundhed, blev hverken nævnt i oversigtartikler eller identificeret ved en ekstra litteratursøgning.

Anbefalinger

Der gives ikke nogen specifikke anbefalinger for dette punkt.

Kilder

1

E. coli-resistente dyr

Hvad ved vi?

Der er identificeret kandidater til gener som koder for modtageligheden overfor tilhæftningen af F4- og F18-fimbrietyper, og det er muligt at avle grise, der er resistente for specifikke fimbrietyper. For eksempel har et dansk avlsprogram avlet mod en allel af MUC4 genet, der er associeret med resistens mod bestemte ETEC F4 varianter. En stor svaghed er, at der sandsynligvis vil opstå nye *E. coli* stammer med ændringer i sine fimbriae, og disse vil således vokse frem i de resistente grise. Ingen af oversigtsartiklerne rapporterede om avlslinjer med generaliseret resistens mod *E. coli*-associeret fravænningsdiarré.

Anbefalinger

Der gives ikke nogen specifikke anbefalinger for dette punkt.

Kilder

2,3

Management før fravænning

Alder ved fravænning

Hvad ved vi?

Tidlig fravænning kan have konsekvenser for grisenes tarmudvikling, sundhed og produktivitet på lang sigt. Fravænning før 21 dage er forbudt og kan ikke anbefales baseret på den tilgængelig evidens. Tarmmorfologien påvirkes mere markant af fravænning før 28 dage, og faldet i daglig tilvækst efter fravænning er mere prominent ved fravænning før 28 dage. Oversigtsartiklerne anbefalede generelt at undgå fravænning før 26-28 dage. Det kan muligvis være fordelagtigt at øge fravænningsalderen yderligere, men evidensen, der kan understøtte dette, er ikke entydig.

Anbefalinger

✓ Fravæn ikke grise yngre end 28 dage. Det kan overvejes at øge fravænningsalderen yderligere.

Kilder

1,2,4–9

Kropsvægt ved fravænning

Hvad ved vi?

Kropsvægten ved fravænning kan prædiktere, hvordan en gris præstere på lang sigt. Fravænningsvægten kan kondenseres til at være en funktion af kuldstørrelse, fødselsvægt, management i pattegriseperioden, genetik og fravænningsalder. Således kan fravænningsvægt ses som en indirekte markør (proxy) for en række specifikke beskyttende/risiko faktorer for fravænningsdiarré, fx råmælksindtag eller fravænningsalder. I dette videnskabelige resumé gives, der specifikke anbefalinger for de punkter relateret til fravænningsdiarré som fravænningsvægten er en proxy for, og ikke hvordan fravænningsvægten kan øges. I den citerede kilde kan der findes deltaljerede anbefalinger om, hvordan fravænningsvægten kan øges. Fravænningsvægten kan dog stadig være relevant at overvåge for indirekte at måle på punkterne vedrørende management før fravænning og fravænningsalder.

Anbefalinger

Der gives ikke nogen specifikke anbefalinger for dette punkt.

Kilder

10

Råmælksindtag

Hvad ved vi?

Råmælksindtag er vigtigt for tarmudviklingen efter fødsel. Hvilken betydning immuniteten, der overføres via råmælken, har for fravænningsdiarré, er ikke beskrevet grundigt i litteraturen. Lavt råmælksindtag kan dog føre til lav fravænningsvægt og forøge risikoen for andre tilstande efter fravænning så som dødelighed og post-weaning multisystemic wasting syndrome (PMWS). Splitmalkning hjælper med at sikre tilstrækkelig råmælk til grise der er i risiko for at modtage for lidt.

Anbefalinger

- ✓ Praktiser splitmalkning i kuld med grise i risiko for at få for lidt råmælk, fx små grise og grise med intrauterint væksthæmningssyndrom (IUGR) født i store kuld.
- ✓ Udfør splitmalkningen så tidligt som muligt efter faring, og ikke senere en 24 timer efter faring, fx i 2 timer per gang.
- ✓ Svagfødte grise kan have gavn af at få hjælp til at finde yveret umiddelbart efter de er blevet født.
- ✓ Svagfødte grise kan have gavn af at modtage udmalket råmælk som per oralt supplement.

Kilder

1,2,6,11–16

Fødselsvægt

Hvad ved vi?

Lav fødselsvægt, eventuelt sammenhængende med intrauterint væksthæmningssyndrom (IUGR), er en risikofaktor for lavt råmælksindtag, dødelighed i dieperioden, lav fravænningsvægt og andre uønskede følgevirkninger i dieperioden. IUGR grise har en langsommere udvikling af mave-tarmsystemet og en anderledes mikrobiota end grise uden IUGR. Ingen af de gennemgåede oversigtsartikler havde som formål at undersøge fødselsvægtens betydningen for parametre efter fravænning, men vi fandt tre originale forskningsartikler, der rapporterede at lav fødselsvægt hænger sammen med reduceret tilvækst efter fravænning og et forringet inflammatorisk respons samt nedsat absorptionskapacitet og antioxidant-egenskaber i tarmen. Et observationsbaseret feltstudie rapporterede at forekomsten af fravænningsdiarré var forøget hos grise med lav fødselsvægt, mens to andre studier præsenterede uklare resultater for effekten af IUGR på den gennemsnitlige fæcesscore efter fravænning. Andelen af grise, der fødes med lav fødselsvægt, kan fx påvirkes gennem fodringen af søerne under drægtigheden eller ved at vælge søer eller avlslinjer/racer med en mindre kuldstørrelse.

Anbefalinger

- ✓ Minimér andelen af grise der fødes med lav fødselsvægt og IUGR.

Kilder

9,10,16–20

Fodring i dieperioden

Hvad ved vi?

Fodring med kornbaseret tørfoder i dieperioden forebygger formodentlig fravænningsdiarré ved at tilvænne fordøjelsen til fast foder, dvs. stimulere produktionen af enzymer og tolerance mod antigener i foderet. Det er uklart, om og hvordan fodring i dieperioden påvirker tarmmikrobiotaen. Samtidig er evidensen for effekten af at tildele pattegrisefoder på forekomsten af fravænningsdiarré ikke entydig. For at have en effekt skal pattegrisefoderet ikke blot tildeles; det skal også spises, og helst i store mængder. En af kilderne foreslår, at grise ikke bør fravænnes før de har indtaget omkring 600 g tørfoder, hvilket dog ofte vil kræve en betydelig forøgelse af fravænningsalderen i mange produktionssystemer. Senere fravænning tillader øget foderindtag før fravænning, og ved typiske fravænningsaldre (fx 28 dage) vil det ikke være alle grise, der er begyndt at spise af pattegrisefoderet endnu, og de som gør vil også kun spise små mængder. Det er uklart, hvornår man skal begynde at tildele pattegrisefoder og artiklerne gav modstridende anbefalinger. Mens nogen rapporterede, at det kunne være tilstrækkeligt at tildele foder i 2-3 dage umiddelbart før fravænning for at opnå en den potentielle effekt, anbefalede andre længere perioder. Ikke desto mindre spiser stort set ingen grise foder før dag 14 efter faring og foderindtaget vil som regel være begrænset før dag 21 efter faring. Måden foderet tildeles på, fodersammensætningen og foderets form har betydning for, hvor meget grisene spiser.

Anbefalinger

- ✓ Tildel foder til pattegrisene i dieperioden med et mål om at mindst 600 g foder indtages per gris før fravænning.
- ✓ Fokusér på at maksimere andelen af grise som spiser og mængden de spiser.
- ✓ Fodertildeling før dag 14 før efter fødsel har muligvis ingen effekt.
- ✓ Fodertildeling før dag 21 efter fødsel har sandsynligvis kun en mindre effekt.
- ✓ Vådfoder/opblødt tørfoder anbefales.
- ✓ Hvis der fodres med piller, bør det være store og bløde piller.
- ✓ Foderets fordøjelighed og kompleksitet bør være høj, mens energitætheden bør være moderat.
- ✓ Anvend fodertrug, der stimulerer legeadfærd, og fodertrug, som er nemme at finde og tilgå for grisene og tillader social ædeadfærd.

Kilder

1,5,10,13,15,21–23

Supplerende fodring med mælkeerstatning i dieperioden

Hvad ved vi?

Supplerende mælkefodring til pattegrise blev citeret for at øge overlevelsen og tilvæksten før fravænning, i hvert fald for grise med lav fødselsvægt. Et studie blev citeret for at dokumentere øget tilvækst efter fravænning, og et andet studiet har vist en reduceret forekomst af diarré før fravænning og reduceret forekomst af fravænningsdiarré. Dog bør somælk forblive pattegrisesenes primære ernæringskilde. Supplerende mælk bliver hovedsageligt spist af små grise, der ligger hos søer med dårlig mælkeproduktion og/eller store kuld. Der vil sandsynligvis være forskel på i effektiviteten af supplerende mælkefodring mellem producenter. Det er uklart om tilsætning af funktionelle ingredienser har gavnlige effekter.

Anbefalinger

✓ Tildel supplerende fodring med mælkeerstatning i dieperioden hos søer med store kuld og/eller dårlig mælkeproduktion.

Kilder

5,10,15

Vaccination af søer

Hvad ved vi?

Vaccination af søer forud for farring stimulerer produktionen af specifikke antistoffer, som beskytter pattegrisene beskyttelse gennem råmælken og somælken. Forskellige typer at ETEC-vacciner er markedsført af en række producenter. Ingen af de inkluderede oversigtsartikler diskuterede betydning af maternel immunitet for fravænningsdiarré eller sundheden og produktiviteten efter fravænning generelt.

Anbefalinger

Der gives ikke nogen specifikke anbefalinger for dette punkt.

Kilder

11,24

Antibiotikabehandling i dieperioden

Hvad ved vi?

Antibiotikabehandling i dieperioden påvirker tarmens mikrobiota, og effekterne deraf er blevet sporet i mindst fem uger, dvs. også efter fravænning. Immunologiske processer samt fordøjelsen og optaget af næringsstoffer påvirkes også. Om, og hvordan dette påvirker grisenes præstation og kliniske parametre efter fravænning blev ikke nævnt i oversigtsartiklerne, og en søgning efter yderligere litteratur afslørede ikke nogle studier, der havde målt på sådanne parametre.

Anbefalinger

Der gives ikke nogen specifikke anbefalinger for dette punkt.

Kilder

1

Delfravænning af kuld

Hvad ved vi?

Ingen af oversigtsartiklerne beskæftigede sig med delfravænning af kuld, hvor et kuld fravænnes i to stadier for at give de mindste grise noget eksklusiv ekstratid med soen. Vi kunne ikke finde relevante fagfællebedømt forskningsartikler.

Anbefalinger

Der gives ikke nogen specifikke anbefalinger for dette punkt.

Management under fravænningen

Gradvis fravænning

Hvad ved vi?

Der er blevet udviklet staldsystemer, hvor søerne selv kan gå til og fra pattegrisene og systemer, hvor flere kuld går sammen evt. kun med én so, for blandt andet at muliggøre en mere gradvis fravænning produktion. Nærværende resume fokuserer på forandringer, der er mulige inden for eksisterende staldsystemer; se de citerede oversigtsartikler, hvis en større produktionsomlægning overvejes.

Managementpraksissen "afbrudt diegivning" er en anden måde at opnå en mere gradvis fravænning. Det indebærer en periodevis adskillelse af pattegrisene og soen i slutning af den planlagt dieperiode. Dette vil øge indtaget af fast føde før og efter fravænning, øge tilvæksten efter fravænning og have en positiv på tarmmorfologien efter fravænning. Dog øger afbrudt diegivning ikke andelen af grise, som er "tørfoderspisere", men til gengæld øges den mængde foder som "tørfoderspiserne" indtager markant. En effekt, der irrelevant for forebyggelsen af fravænningsdiarré, men værd at notere sig, er at tre dages separation henover natten (16 timer) i den fjerde laktationsuge vil inducere brunst og succesfuld befrugtning med en frekvens, som kan være rimelig ud fra en produktionsøkonomisk betragtning. Ulempen ved periodevis afbrudt diegivning er, at det kræver ekstra arbejdskraft og at tilvæksten i dieperioden kan falde. Sidstnævnte vil dog i nogen grad kompenseres af at grisene vil præstere bedre i den første uge efter fravænning. Oversigtsartiklerne var ikke enige om, hvorvidt praksissen forårsager ekstra stress for pattegrisene og soen; artiklen som påpegede, at det kunne være et problem, citerede dog ikke nogen studier. Den direkte effekt på fravænningsdiarré var ikke rapporteret i nogen artikler.

En mere gradvis fravænning kan også opnås indirekte ved at fravænne grisene (markant) senere (fx 6-8 uge). I ældre studier, hvor denne fravænningsalder var almindelig praksis i indendørsproduktion blev 50-80% af energiindtaget umiddelbart før fravænning dækket af fast foder. Modsat indtager grisene i nutidens produktionsformer ganske lidt foder og lever næsten udelukkende af somælken, når de adskilles fra soen.

Konklusionen er, at de mulige anbefalinger generelt vil kræve strategiske beslutninger (fx opføre ny stald eller skifte staldinventar). Dette inkluderer en anbefaling om at praktisere afbrudt diegivning i kombination med en dieperiode på >33 dage, hvilket dog kan være muligt i nogle tilfælde indenfor eksisterende systemer.

Anbefalinger

Der gives ikke nogen specifikke anbefalinger for dette punkt.

Kilder

9,13,25

Håndtering under fravænning/ flytning af grisene

Hvad ved vi?

Det er veletableret, at et fravænningsinduceret stressrespons kan svække immunforsvaret og tarmfunktionaliteten og således øge modtageligheden for fravænningsdiarré. Når grisene flyttes fra farestierne til klimastierne eller en transportvogn vil det hypotetisk være en kilde til stress, og betydningen af proceduren vil sandsynligvis afhænge af, hvordan den udføres. Imidlertid fandt vi hverken oversigtsartikler eller originale forskningsstudier af effekten af håndteringen under flytningen af grisene. Hos slagtegrise er sådanne effekter dog veldokumenterede, og der findes en betydelige evidens om, hvordan grisene flyttes på den nemmeste og mindst stressende måde. Vi afgjorde, at det var rimeligt at generalisere anbefalingerne til fravænnede smågrise.

Anbefalinger

- ✓ Afstanden som grisene flyttes/drives bør være så kort som muligt
- ✓ Undgå at blande grise med ukendte dyr, eller sammenbland dem kun med de grise de skal gå sammen med i klimastalden.
- ✓ Flyt grise i små grupper, fx et kuld af gangen.
- ✓ Sørg for at gangarealerne er veloplyste og at grisene ikke presses til at gå fra oplyste til mørke rum.
- ✓ Undgå støj i de gangarealer som grisene drives igennem.

Kilder

7,9,26,27

Transport

Hvad ved vi?

Litteraturen domineres af studier af ældre grise og vi valgte at tage dette i betragtning sammen med den begrænsede litteratur om nyligt fravænnede smågrise. Transport kan føre til et akut stressrespons i fravænnede grise, som kommer i tillæg til den stress, der påføres af fravænningen i sig selv. Selve håndteringen, når grisene drives ombord på og af transportvognen, og sammenblanding med ukendte individer er betydningsfulde transportassocierede stressfaktorer. Lange transporttider kan også forårsage dehydrering og faste. Dog vil fasten og vandmanglen som forårsages af transporter på mindre end 6-8 timer sjældent påføre ekstra skade for tidligt fravænnede grise; de vil som regel også have lange fasteperioder, når de fravænnes direkte ind i en klimasti. Forholdene under transporten ser også ud til at være af betydning, og både årstiden og pladsforhold er afgørende for hvilken indvirkning transporten har på grisene. Fravænnede grise er særligt følsomme over for varmestress under længerevarende transport, og kolde temperaturer bør også undgås. Ingen af oversigtsartiklerne nævnte studier, der havde set på den direkte effekt af transport på fravænningsdiarré.

Anbefalinger

- ✓ Grisene skal have mindst 0.08 m^2 under transporten.
- ✓ Sørg for tilstrækkelig temperaturregulering og beskyttelse mod træk under transporten.
- ✓ Gennemgå håndteringen af grisene, praksis når grisene drives ombord og af transportvognen, og designet af udstyr og faciliteter; kan der gøres noget for at minimere den stress som påføres grisene?
- ✓ Minimér sammenblanding med fremmede grise før, under og efter transport.

Kilder

28,29

Fravænning i farestien

Hvad ved vi?

Vi fandt ikke noget relevant litteratur til dette punkt.

Anbefalinger

Der gives ikke nogen specifikke anbefalinger for dette punkt.

Management efter fravænning

Belægningsgrad

Hvad ved vi?

Høj belægningsgrad er en social stressfaktor for grisene efter fravænning, som fører til nedsat præstation og forøget sygdomsforekomst. Vi fandt ikke nogen studier, der havde målt effekten af belægningsgrad på fravænningsdiarré.

Anbefalinger

- ✓ Mindst 0.34 m^2 gulvareal per gris anbefales.

Kilder

8,22,26,30,31

Gruppestørrelse per sti

Hvad ved vi?

Begrænset evidens indikerer, at der ikke er nogen klar effekt af gruppestørrelse på forekomsten af fravænningsdiarré. I oversigtsartikler er en del studier af effekten af gruppestørrelse på grises præstation efter fravænning blevet gennemgået og metaanalyseret. Der er en ikke-lineær effekt på tilvækst og foderindtag. Et svagt fald i tilvækst og foderindtag ses når gruppestørrelsen øges fra 10 grise op mod 100 grise. Imidlertid ses der klare forbedringer i tilvækst og foderindtag, når gruppestørrelsen holdes under 10 grise. Forekomsten af aggressiv adfærd efter sammenblanding ser ikke ud til at stige med forøget gruppestørrelse.

Anbefalinger

Der gives ikke nogen specifikke anbefalinger for dette punkt.

Kilder

32–34

Sortering af grisene i stierne

Hvad ved vi?

Sammenblanding med fremmede grise er en social stressfaktor for grisene, der fører til agonistisk adfærd og øger smitten med patogener. Dette sænker tilvæksten og foderindtaget, svækker immunforsvaret, og resulterer i bidsår og andre uønskede følgevirkninger. Således er den indirekte sammenhæng til tarmsundhed efter fravænning klar, men der var ikke rapporteret studier af sammenhængen med forekomsten af fravænningsdiarré. Kuldvis opstaldning forbygger ovenstående. Der er ikke overbevisende evidens for, at grise, som er sorteret efter kropsvægt, præsterer bedre end grise, der sorteres vilkårligt; faktisk er det modsatte muligvis tilfældet. Dog kan målrettet fodring være et argument for at sortere grisene på denne måde. Det er rapporteret, at sortering efter køn kan reducere forekomsten af slåskampe og aggressiv adfærd efter sammenblanding. Rode- og beskæftigelsesmateriale kan reducere forekomsten af aggressiv adfærd. Ved at blande grisene med fremmede individer fra andre kuld før fravænning, særligt når grisene er 7-12 dage gamle, kan grisenes sociale egenskaber forbedres og således også deres evne til at håndtere sammenblanding efter fravænning. Dette vil dog ofte kun lade sig gøre, hvis der bygges nye staldsystemer, og det kan have andre ulemper (fx risiko for smitteoverførsel).

Anbefalinger

- ✓ Sammenbland ikke grise, der stammer fra forskellige sobesætninger.
- ✓ Sorter grisene i kuld, fx hold 2-3 hele kuld i stier med 30 stipladser.
- ✓ Hvis sammenblanding er uundgåelig, kan sortering efter køn være gavnligt.
- ✓ Hvis sammenblanding er uundgåelig, kan social stress forårsaget af sammenblanding minimeres ved at tildele mere rode-og beskæftigelsesmateriale.
- ✓ At sortere grisene efter størrelse er muligvis spild af arbejdskraft; foretag en evaluering af hvilke fordele, der opnås ved sorteringen for at retfærdiggøre denne praksis.
- ✓ Hvis der anvendes midlertidige smågrisestier (fx “babycontainere”), så bør gruppekompositionen i stierne bevares fremfor at grisene sammenblandes på nyt i de permanente klimastier.

Kilder

1,8,9,26,30,34

Rode- og beskæftigelsesmateriale

Hvad ved vi?

Hvis der mangler rode- og beskæftigelsesmateriale vil fravænnede grise oftere udføre en række unormale adfærdstyper. Et studie blev citeret for at vise, at tildeling af rodemateriale reducerede forekomsten af fravænningsdiarré og øgede tilvæksten hos fravænnede grise. Baseret på den tilgængelige evidens kunne vi dog ikke komme med specifikke anbefalinger omkring, hvor meget berigelse, der bør tildeles for, at det er tilstrækkeligt.

Anbefalinger

- ✓ Tildel rode- og beskæftigelsesmateriale (fx halm) i tilstrækkelige mængder.

Kilder

23,35

Rumtemperatur og luftflow

Hvad ved vi?

Kølige temperaturer og træk øger forekomsten af fravænningsdiarré. For høje temperaturer kan reducere foderindtaget og tilvæksten. Fluktuationer i temperaturen sænker tilvæksten og øger forekomsten af fravænningsdiarré.

Anbefalinger

- ✓ Hvis der ikke forefindes lokale varmekilder (fx overdækning med varmelamper eller overdækning og gulvvarme), bør temperaturen i stalden være 26-28 grader C de første 14 dage efter fravænning.
- ✓ Temperaturer ned til 23 grader C er acceptable, når der forefindes lokale varmekilder (fx overdækning med varmelamper eller overdækning og gulvvarme).
- ✓ Sørg for at temperaturen er stabil og konstant i hele rummet.
- ✓ Sørg for at grisene ikke udsættes for træk (kølige luftstrømme på > 0.2 m/s).

Kilder

2,8,31,34,36,37

Luftkvalitet

Hvad ved vi?

Koncentrationen af støvpartikler, bakterier og toksiner, ammoniak (NH_3), hydrogensulfid, og kuldioxid (CO_2) i luften i stalden har betydelig effekt på foderindtaget og tilvæksten efter fravænning. Sammenhængen kan relatere sig til den markante effekt på immunforsvaret og sygdomme i luftvejene. En søgning efter oversigtsartikler, der diskuterer effekten på mave-tarmsundheden gav ikke nogen resultater, men vi fandt er observationsbaseret feltstudie. De fandt en association mellem luftkvalitetsindeks og forekomsten af fravænningsdiarré. Dog var indekset en kombination af mål for træk og koncentrationen af forskellige gasarter i stalden. God luftkvalitet kan sikres vha. passende ventilation, grundig rengøring af stierne og ved at sænke belægningsgraden, så der er et luftrum (m^3/gris) på mindst 0,0118*kropsvægt(kg)+1,82 (dvs. 1,9144 m^3/gris for 8 kg grise). Der blev ikke foreslået grænseværdier for alle relevante substanser i den litteratur vi har gennemgået, så nogle af værdierne er hentet fra et bogkapitel.

Anbefalinger

- ✓ Sørg for god luftkvalitet dvs. NH_3 koncentration under 5 ppm, CO_2 koncentration under 1500 ppm, endotoksin koncentration under 1 µg/m^3 og levedygtige bakterier under 50 000 CFU/m^3 luftrum samt en lav koncentration af støvpartikler.

Kilder

8,31,36,38

Foder- og vandtildelingsstrategi

Plads ved fodertruget

Hvad ved vi?

Tilstrækkelig plads ved fodertrugene minimerer konkurrencen om foder og giver mulighed for at æde i grupper, hvilket opfylder grisenes præferencer og giver mulighed for social indlæring af ædeadfærd. Når pladsen ved fodertrugene øges, vil grisene begynde at æde tidligere og de øger generelt foderoptagelsen og tilvæksten. Et observationsbaseret feltstudie har rapporteret, at risikoen for fravænningsdiarré var højere i svinebesætninger med begrænset plads ved fodertrugene. Baseret på den tilgængelige evidens, kunne vi ikke komme med specifikke anbefalinger til den optimale plads ved fodertrugene per gris, men vi foreslår at alle grise bør kunne spise på samme tid.

Anbefalinger

- ✓ Sørg for, at der er nok plads ved fodertrugene til at alle grise kan spise på samme tid
- ✓ Hvis ovenstående anbefaling ikke opfyldes, kan der indsættes ekstra fodertrug midlertidigt de første 14 dage efter fravænning, så anbefaling opfyldes i denne risikoperiode.

Kilder

2,22,34

Fodringsregime

Hvad ved vi?

Foder kan gives enten ad libitum eller restriktivt med forskellige fodringsplaner. Fra både eksperimentelle og observationsbaserede feltstudier er der god evidens for, at restriktiv fodring reducerer forekomsten af fravænningsdiarré sammenlignet med ad libitum fodring. Tilvæksten vil sandsynligvis blive reduceret i perioden med restriktiv fodring, men dette kan muligvis afbødes af en kompensatorisk vækst, når der skiftes over til ad libitum fodring senere i grisenes liv.

Anbefalinger

- ✓ Praktiser restriktiv fodring i de første 14 dage efter fravænning.
- ✓ Giv 4-8 måltider per dag i stedet for f.eks. 1-2 måltider per dag.

Kilder

2,8

Gulv-/måttefodring

Hvad ved vi?

Foder kan tildeles på gulvet eller på en måtte i stier med fuldspaltegulv. Den refererede oversigtsartikel havde kun fundet en begrænset evidens omkring denne praksis, og den konkluderede, at de tilgængelige data var utilstrækkelige til at dokumentere effektiviteten af gulv-/måttefodring. Imidlertid havde to undersøgelser rapporteret et fald i dødeligheden efter fravænning ved gulvfodring. Måttefodring stimulerer ædeadfærden den første dag efter fravænning, men fører også til for øget foderspild, især efter den tredje dag efter fravænning.

Anbefalinger

✓ Giv foder på gulvet de første 1-3 dage efter fravænning.

Kilder

34

Grødfoder

Hvad ved vi?

"Grødfoder" tilberedes ved at opbløde foderpiller eller melfoder i vand (f.eks. 1:1 eller 1:2). Flere undersøgelser har dokumenteret, at grise, der er fodres med grødfoder, æder mere og vokser hurtigere i perioden efter fravænning, og at grødfodring inducerer højere tyndtarmsvilli og øger mængden af mælkesyreproducerende bakterier i tarmen. Imidlertid rapporterede ingen undersøgelser om effekten på forekomsten af fravænningsdiarré, og en af oversigtsartiklerne konkluderede, at de tilgængelige data var utilstrækkelige til at dokumentere om grødfodring var effektivt. Grødfodring kræver ekstra arbejdskraft. Ved at tildele en mindre andel af det fulde næringsstofbehov som grød i mindre måltider, minimerer man muligvis foderspild (da hele måltidet hurtigt spises op), og det sikres, at grisene kommer i gang med at indtage tørfoder. Der skal holdes god hygiejne i fodertrugene.

Anbefalinger

✓ Suppler tørfoderet med 3-4 små grødmåltider per dag de første 7-14 dage efter fravænning samtidig med, at der holdes god hygiejne i fodertrugene.

Kilder

8,34,37

Foderets partikelstørrelse

Hvad ved vi?

Formaling og hydrotermisk forarbejdning af foderet giver finere partikelstørrelser og øger dermed fordøjeligheden, men dette er klart associeret med mavesår, og et mere groft foder kan være gavnligt for tarmsundheden. Fravænnede pattegrise foretrækker groft formalet foder. En begrænset mængde evidens tyder på, at tildeling af groft foder til unge grise sænker mavens pH, forlænger tiden som foderet opholder sig maven, forebygge kolonisering af Salmonella og ETEC i ileum og øger dybden af tyktarmskrypterne og fermenteringen i tyktarmen og dermed produktionen af organiske syrer; dvs. groft foder begrænser faktorer, der er involveret i årsagssammenhængen for fravænningsdiarré. En undersøgelse rapporterede også, at inklusion af 4% groft formalet hvedeklid reducerede forekomsten af fravænningsdiarré hos grise, der blev eksperimentelt inficeret med ETEC, sammenlignet med grise fodret med fint malet hvedeklid. Billige foderstoffer med lavt energiindhold kan anvendes ved tilsætning af grove partikler for at minimere tabet som følge af dårlig fordøjelighed for de grove partikler.

Anbefalinger

- ✓ Undgå partikelstørrelser <0,4 mm
- ✓ Inkludér partikelstørrelser 0,5-1,6 mm
- ✓ Inkludér partikelstørrelse >1,6, men vær samtidig opmærksom på, at det kan betyde en reduceret fordøjelighed.
- ✓ Tilføj store partikler lavet af lavenergifoder med fordøjelige fibre.

Kilder

15,34,39–41

Foderets fysiske form

Hvad ved vi?

Flere undersøgelser dokumenterer, at den daglige tilvækst er højere for pelleteret foder end for melfoder. Imidlertid er væksten af Salmonella og *E. coli*/ETEC i tarmen lavere for grise fodret med melfoder end pelleteret foder, og en undersøgelse rapporterede en øget risiko for PWD i svinebesætninger, der bruger pelleteret foder. Unge smågrise foretrækker store piller, og hårde piller er svære for dem at tygge/spise.

Anbefalinger

- ✓ Brug hellere melfoder end foderpiller.
- ✓ Hvis der bruges foderpiller, så brug bløde og store (fx Ø = 12mm) piller.

Kilder

8,34,37,39

Vandforsyning

Hvad ved vi?

Nem og fri adgang til vand vil øge vandindtaget, hvilket er forbundet med øget foderindtag og tilvækst efter fravænning. Sammenhængen med forekomsten af fravænningsdiarré er ikke helt klar, men let adgang til vand er især vigtig for grise, der lider af diarré. Grise foretrækker vandkilder placeret i nærheden af foderautomaten frem for ud mod gangen eller bagerst i stien. Det tager længere tid for nyfravænnede grise at lære at drikke af drikkenipler end af skåle, især hvis grisene ikke allerede er bekendt med drikkenipler fra deres farestier. Grise spilder også mindre vand, når de drikker fra skåle. Et for lavt vandtryk kan reducere vandindtaget.

Anbefalinger

- ✓ Sørg for mindst en vandkilde per 10 smågrise.
- ✓ Indsæt et ekstra vand i perioden umiddelbart efter fravænning for at opfylde grisenes behov i denne risikoperiode.
- ✓ Skåle anbefales frem for drikkenipler til nyfravænnede grise.
- ✓ Placér vandkilder i nærheden af fodringssteder.
- ✓ Hvis vandet ikke tildeles i en skål med et stort reservoir, skal det leveres med en hastighed på mindst 0,45 l/min.
- ✓ Vand bør også være tilgængeligt for grise, som fodres med vådfoder.

Kilder

8,34,37,42

Smittebeskyttelse og smittehåndtering

Rengøring før indsættelse

Hvad ved vi?

Koncentrationen af patogener og forurenende partikler, der aktiverer immunsystemet, i staldmiljøet, kan reduceres drastisk ved at rengøre sektioner ordentligt inden indsættelse af nye grise. Fravænning til rene miljøer er associeret med øget præstation, delvist på grund af direkte eller indirekte effekter på tarmsundheden. Et hygiejnogram (hvor kimtallet bestemmes i prøver taget fra stimiljøet) kan bruges til at kvalitetssikre af rengøringsproceduren.

Anbefalinger

- ✓ Vask, desinficer og udtør stierne inden der indsættes et nyt hold grise.

Kilder

8,22,31,36,43

Alt ind/ alt ud praksis

Hvad ved vi?

Alt ind/alt ud-praksis indebærer, at et hold af nyfravænnede smågrise indsættes i et rengjort, tomt rum på samme tid, og at der ikke tillades flytninger eller indsættelse af flere dyr før hele rummet er tømt igen. Dette bryder smitteveje og reducerer derved forekomsten af fravænningsdiarré (og andre sygdomme) og øger generelt grisenes sundhed og produktivitet.

Anbefalinger

- ✓ Praktiser strikt alt-ind/alt-ud management.
- ✓ Vær opmærksom på, at brud på alt-ind/alt-ud praksis kan ske ved brug af midlertidige stier (fx babycontainere) eller supplerende indsættelse af grise fra ammesøer. Minimer omfanget og virkningen af disse brud.

Kilder

8,22,31,44

Personale og deres tøj som potentielle fomitter

Hvad ved vi?

Besætningspersonale og andre personer, der besøger besætningen, kan agere fomitter (”passive smittebærere”) og overføre smitsomme patogener mellem sektioner, stier og grise. Forskellige foranstaltninger kan bryde disse smitteveje. Specifikt er støvler, tøj og hænder/handsker i høj risiko agere fomit.

Anbefalinger

- ✓ Der bør være særskilt tøj til rådighed for de forskellige staldafsnit i besætningen.
- ✓ Anvend handsker og skift dem ofte og/eller vask hænder med jævne mellemrum.
- ✓ Anvend fodbade mellem staldafsnit: Rengør først støvlerne med sæbevand og børste. Træd derefter op i baljen med desinfektionsmiddel og behold støvlerne i opløsningen i tilstrækkelig tid i henhold til producentens instruktioner (fx mindst 30 sek. for Virkon^{TM} S). Skift opløsningen helst hver dag eller mindst hver tredje dag.
- ✓ Alternativt til fodbad: brug forskellige sæt fodtøj hvert staldafsnit.
- ✓ Forslagsvis kan foranstaltningerne, der anbefales mellem forskellige staldafsnit, anvendes før adgang til sektioner, hvor der holdes nyfravænnede grise.

Kilder

43

Arbejdsrutine fra de yngste til de ældste grise

Hvad ved vi?

Nyfravænnede grise vil være mere følsomme over for bestemte patogener end ældre grise. Overførsel af disse patogener kan forebygges ved at implementere arbejdsrutiner, hvor unge grise ikke besøges efter kontakt med ældre grise.

Anbefalinger

- ✓ Besøg og udfør alle procedurer i sektioner med nyfravænnede smågrise som det første i løbet af arbejdsdagen i smågrisebesætningen.
- ✓ Hvis det er nødvendigt at gå ind i en sektion med nyfravænnede smågrise senere på dagen, kan risikoen for smitteintroduktion minimeres ved at følge anbefalingerne for ”*Personale og deres tøj som potentielle fomitter*” (se ovenfor).
- ✓ Gå ikke ind i smågrisestalden efter at have besøgt en slagtesvinestald.

Kilder

44

Renhold af potentielle fomitter

Hvad ved vi?

Redskaber og materialer, som anvendes i besætningen, kan fungere som fomitter (passive smittebærere). Det kan undgås at redskaber flyttes mellem sektioner ved at have særskilte redskaber til hver sektion, og disse kan farvekodes.

Anbefalinger

- ✓ Sørg for, at der er særskilte materialer og redskaber til rådighed i hver sektion i smågrisestalden.
- ✓ Redskaber og materialer, som deles mellem sektioner og ugehold, (fx drivbrædder, legetøj, trug osv.), bør rengøres før de anvendes i et nyt ugehold/en anden sektion.

Kilder

43,44

Renhold af transportvogn

Hvad ved vi?

I multisitesystemer transporteres fravænnede smågrise fra sobesætningen til smågrisebesætningen. Transportvognene kan være kontamineret med patogener fra tidligere ugehold. Det udgøre en yderligere risiko, hvis den samme transportvogn også anvendes til transport af ældre dyr (f.eks. til slagteriet). Rengøring, desinfektion og udtørring af transportvognene forhindre smitteoverførsel.

Anbefalinger

- ✓ Brug (eksterne) transportører som følger certificerede vaskeprogrammer, fx SPF-godkendte transportører.
- ✓ Hvis der anvendes egen transportvogn, skal der iværksættes en passende rengørings og desinfektionsprogram, som gennemføres efter hver transport.
- ✓ Brug aldrig en ikke-rengjort transportvogn, som har transporteret ældre dyr.

Kilder

43,44

Rent drikkevand

Hvad ved vi?

Drikkevand kan være forurenet med patogener. Enten kan vandet, der kommer fra anlægget eller en lokal brønd, være af dårlig kvalitet, eller også kan systemerne i besætningen være forurenet med biofilm. I Danmark leveres vandet ofte fra offentlige vandværker, hvor vandkvaliteten jævnligt kontrolleres. Hvis der anvendes en lokal brønd, bør vandkvaliteten kontrolleres mindst en gang årligt. Dårlig vandkvalitet skal udbedres.

Anbefalinger

- ✓ Brug vand fra offentlige vandværker.
- ✓ Alternativ til ovenstående anbefaling: Foretag selv kontrol af vandkvaliteten mindst en gang om året.
- ✓ Hvis vandkvaliteten er dårlig, skal du korrigere problemet (ved at bruge en passende mekaniske, fysiske eller kemiske behandlinger).
- ✓ Vandsystemer (tanke, rør osv.) i besætningen bør rengøres og desinficeres regelmæssigt.

Kilder

43,44

Management af specifikke patogener

Vaccination mod ETEC

Hvad ved vi?

Der er pt markedsført én levende *E. coli*-vaccine med F4ac- og F18ac-stammer i Danmark (COLIPROTEC® F4/F18). Vaccinen kan gives oralt både før eller efter fravænning. Det kan være svært at finde det bedste tidspunkt at administrere vaccinen, da F18-receptorerne først udtrykkes, når grisene er ca. 17 dage gamle. Derudover kan immuniteten, der kontinuerligt overføres med somælk, hæmme virkningen af vaccinen hos pattegrise, mens akut stress induceret af fravænningen kan svække immunkompetencen hos grise efter fravænning. Kliniske effekter af vaccinen kan kun forventes, hvis den administreres rettidigt, og hvis fravænningsdiarréen er forårsaget af ETEC-stammer, der matcher den immunitet som vaccinen inducerer.

Anbefalinger

- ✓ Bekræft tilstedeværelsen af ETEC med fimbrie-antigener, der matcher vaccinens, før det overvejes at påbegynde et vaccinationsprogram.
- ✓ Brug den levende orale vaccine i tilfælde af ETEC-associeret fravænningsdiarré.
- ✓ Vaccinér ikke grisene før de er 17 dage gamle.
- ✓ Vaccinér grisene mellem 7 og 21 dage før forventet peak i forekomsten af ETEC-associeret fravænningsdiarré.

Kilder

45–47

Vaccination mod rotavirus

Hvad ved vi?

Vacciner mod rotavirus markedsføres i øjeblikket kun til (immunisering før faring hos) søer, og effekten af denne vaccine på fravænningsdiarré er ukendt. Afprøvning af vacciner til nyfødte grise i praksis har ikke konsekvent kunne vise god effekt. Interaktionen med den laktogene immunitet og mangfoldigheden af rotavirusgrupper og stammer inden for grupperne gør det vanskeligt at producere rotavirusvacciner, der er effektive under praksisforhold.

Anbefalinger

Der gives ikke nogen specifikke anbefalinger for dette punkt.

Kilder

48

Referencer

1. Trevisi P, Luise D, Correa F, Bosi P. Timely Control of Gastrointestinal Eubiosis: A Strategic Pillar of Pig Health. Microorganisms. 2021 Feb 3;9(2).

2. Rhouma M, Fairbrother JM, Beaudry F, Letellier A. Post weaning diarrhea in pigs: risk factors and non-colistin-based control strategies. Acta Vet Scand. 2017 Dec;59(1):31.

3. Rebeka Sinha, Sahoo NR, Kush Shrivastava, Pushpendra Kumar, Salauddin Qureshi, De UK, et al. Resistance to ETEC F4/F18-mediated piglet diarrhoea: opening the gene black box. Trop Anim Health Prod. 2019;51(6):1307–20.

4. Masri SA, Richardson K, Plendl J. Influence of age at weaning and feeding regimes on the postnatal morphology of the porcine small intestine. J Swine Health Prod. 2015;23(4):18.

5. Wensley MR, Tokach MD, Woodworth JC, Goodband RD, Gebhardt JT, DeRouchey JM, et al. Maintaining continuity of nutrient intake after weaning. I. Review of pre-weaning strategies. Transl Anim Sci. 2021 Jan;5(1):txab021.

6. Boyd RD, Zier-Rush CE, Moeser AJ, Culbertson M, Stewart KR, Rosero DS, et al. Review: innovation through research in the North American pork industry. Anim Int J Anim Biosci. 2019 Dec;13(12):2951–66.

7. Moeser AJ, Pohl CS, Rajput M. Weaning stress and gastrointestinal barrier development: implications for lifelong gut health in pigs. Anim Nutr. 2017;3(4):313–21.

8. Kil DY, Stein HH. Invited Review: Management and feeding strategies to ameliorate the impact of removing antibiotic growth promoters from diets fed to weanling pigs. Can J Anim Sci. 2010 Dec;90(4):447–60.

9. Weary DM, Jasper J, Hotzel MJ. Understanding weaning distress. Dybkjaer L, editor. Spec Issue Early Weaning. 2008;110(1/2):24–41.

10. Blavi L, Solà-Oriol D, Llonch P, López-Vergé S, Martín-Orúe SM, Francisco Pérez J. Management and feeding strategies in early life to increase piglet performance and welfare around weaning: a review. Animals. 2021;11(2).

11. Poonsuk K, Zimmerman J. Historical and contemporary aspects of maternal immunity in swine. Anim Health Res Rev. 2018 Jun;19(1):31–45.

12. Alexopoulos JG, Lines DS, Hallett S, Plush KJ. A review of success factors for piglet fostering in lactation. Animals. 2018;8(3):38.

13. Pluske JR. Invited review: aspects of gastrointestinal tract growth and maturation in the pre- and postweaning period of pigs. J Anim Sci. 2016;94(Suppl. 3):399–411.

14. Salmon H, Berri M, Gerdts V, Meurens F. Humoral and cellular factors of maternal immunity in swine. Dev Comp Immunol. 2009 Mar;33(3):384–93.

15. Huting AMS, Middelkoop A, Guan X, Molist F. Using nutritional strategies to shape the gastro-intestinal tracts of suckling and weaned piglets. Animals. 2021;11(2).

16. Everaert N, Cruchten S van, Westrom B, Bailey M, Ginneken C van, Thymann T, et al. A review on early gut maturation and colonization in pigs, including biological and dietary factors affecting gut homeostasis. Spec Issue Feed Ingred Addit Technol Healthy Gut Pigs. 2017;233(Precision Livestock and Nutrition Unit, Gembloux Agro-Bio Tech, TERRA Teaching and Research Centre, University of Liege, Passage de Deportes 2, Gembloux, Belgium.):89–103.

17. Eriksen EØ, Kudirkiene E, Christensen AE, Agerlin MV, Weber NR, Nødtvedt A, et al. Post-weaning diarrhea in pigs weaned without medicinal zinc: risk factors, pathogen dynamics, and association to growth rate. Porc Health Manag. 2021 Dec;7(1):1–19.

18. Che L, Hu L, Zhou Q, Peng X, Liu Y, Luo Y, et al. Microbial insight into dietary protein source affects intestinal function of pigs with intrauterine growth retardation. Eur J Nutr. 2020 Feb 1;59(1):327–44.

19. Novais AK, Deschêne K, Martel-Kennes Y, Roy C, Laforest JP, Lessard M, et al. Weaning differentially affects mitochondrial function, oxidative stress, inflammation and apoptosis in normal and low birth weight piglets. PLOS ONE. 2021 Feb 19;16(2):e0247188.

20. Che L, Zhou Q, Liu Y, Hu L, Peng X, Wu C, et al. Flaxseed oil supplementation improves intestinal function and immunity, associated with altered intestinal microbiome and fatty acid profile in pigs with intrauterine growth retardation. Food Funct. 2019 Dec 11;10(12):8149–60.

21. Pluske JR, Turpin DL, Kim JC. Gastrointestinal tract (gut) health in the young pig. Anim Nutr. 2018 Jun;4(2):187–96.

22. Jayaraman B, Nyachoti CM. Husbandry practices and gut health outcomes in weaned piglets: A review. Anim Nutr. 2017 Sep;3(3):205–11.

23. Widowski TM, Torrey S, Bench CJ, Gonyou HW. Development of ingestive behaviour and the relationship to belly nosing in early-weaned piglets. Appl Anim Behav Sci. 2008 Mar;110(1–2):109–27.

24. Matias J, Berzosa M, Pastor Y, Irache JM, Gamazo C. Maternal Vaccination. Immunization of Sows during Pregnancy against ETEC Infections. Vaccines. 2017 Dec;5(4):48.

25. Downing JA. An opportunity to revolutionise sow management. Anim Prod Sci. 2015;55(11/12):1411–23.

26. Pluske JR, Miller DW, Sterndale SO, Turpin DL. Associations between gastrointestinal-tract function and the stress response after weaning in pigs. Anim Prod Sci. 2019;59(11):2015–22.

27. Goumon S, Faucitano L. Influence of loading handling and facilities on the subsequent response to pre-slaughter stress in pigs. Livest Sci. 2017 Jun;200:6–13.

28. Rioja-Lang FC, Brown JA, Brockhoff EJ, Faucitano L. A review of swine transportation research on priority welfare issues: a Canadian perspective. Front Vet Sci. 2019;6(February):36.

29. Sutherland MA, Backus BL, McGlone JJ. Effects of transport at weaning on the behavior, physiology and performance of pigs. Animals. 2014;4(4):657–69.

30. Proudfoot K, Habing G. Social stress as a cause of diseases in farm animals: Current knowledge and future directions. Vet J. 2015 Oct;206(1):15–21.

31. Gleeson BL, Collins AM. Under what conditions is it possible to produce pigs without using antimicrobials? Anim Prod Sci. 2015 Nov 13;55(12):1424–31.

32. Turner SP, Allcroft DJ, Edwards SA. Housing pigs in large social groups: a review of implications for performance and other economic traits. Livest Prod Sci. 2003;82(1):39–51.

33. Payne HG, Brumm MC, D'Antuono M, Pluske J. R., Williams IH, Moore KL, et al. Review of group size effects on the performance of growing pigs. CAB Rev Perspect Agric Vet Sci Nutr Nat Resour. 2006;1(012):22-pp.

34. Wensley MR, Tokach MD, Woodworth JC, Goodband RD, Gebhardt JT, DeRouchey JM, et al. Maintaining continuity of nutrient intake after weaning. II. Review of post-weaning strategies. Transl Anim Sci [Internet]. 2021 Jan 1 [cited 2021 Aug 3];5(1). Available from: http://academic.oup.com/tas/article/5/1/txab022/6130225

35. Prunier A, Heinonen M, Quesnel H. High physiological demands in intensively raised pigs: impact on health and welfare. Animal. 2010;4(6):886–98.

36. Pluske JR, Kim JC, Black JL. Manipulating the immune system for pigs to optimise performance. Anim Prod Sci. 2018;58(4):666–80.

37. Dong GZ, Pluske JR. The low feed intake in newly-weaned pigs: problems and possible solutions. Asian-Australas J Anim Sci. 2007;20(3):440–52.

38. Madec F, Bridoux N, Bounaix S, Jestin A. Measurement of digestive disorders in the piglet at weaning and related risk factors. Prev Vet Med. 1998 Apr;35(1):53–72.

39. Kiarie EG, Mills A. Role of Feed Processing on Gut Health and Function in Pigs and Poultry: Conundrum of Optimal Particle Size and Hydrothermal Regimens. Front Vet Sci. 2019;6:19.

40. Jansman AJM. Health and functions of the gastrointestinal tract in pigs: Effects of functional ingredients and feed and ingredient processing. J Anim Sci. 2016 Sep;94:12–21.

41. Dirkzwager A, Veldman B, Bikker P. A nutritional approach for the prevention of Post Weaning Syndrome in piglets. Anim Res. 2005;54(3):231–6.

42. Meunier-Salaun MC, Chiron J, Etore F, Fabre A, Laval A, Pol F, et al. Review: Drinking water for liquid-fed pigs. Animal. 2017 May;11(5):836–44.

43. Alarcon LV, Alberto AA, Mateu E. Biosecurity in pig farms: a review. Porc Health Manag. 2021 Jan 4;7(1):5.

44. Filippitzi ME, Kruse AB, Postma M, Sarrazin S, Maes D, Alban L, et al. Review of transmission routes of 24 infectious diseases preventable by biosecurity measures and comparison of the implementation of these measures in pig herds in six European countries. Transbound Emerg Dis. 2018 Apr;65(2):381–98.

45. Laird TJ, Abraham S, Jordan D, Pluske JR, Hampson DJ, Trott DJ, et al. Porcine enterotoxigenic Escherichia coli: Antimicrobial resistance and development of microbial-based alternative control strategies. Vet Microbiol. 2021 Jul;258:109117.

46. Hedegaard CJ, Heegaard PMH. Passive immunisation, an old idea revisited: basic principles and application to modern animal production systems. Vet Immunol Immunopathol. 2016;174(National Veterinary Institute, Technical University of Denmark, Section for Immunology and Vaccinology, The Innate Immunology Group, Bulowsvej 27, DK-1870 Frederiksberg C, Denmark.):50–63.

47. Sun Y, Kim SW. Intestinal challenge with enterotoxigenic Escherichia coli in pigs, and nutritional intervention to prevent postweaning diarrhea. Anim Nutr. 2017 Dec;3(4):322–30.

48. Chattha KS, Roth JA, Saif LJ. Strategies for design and application of enteric viral vaccines. Annu Rev Anim Biosci. 2015;3:375–95.

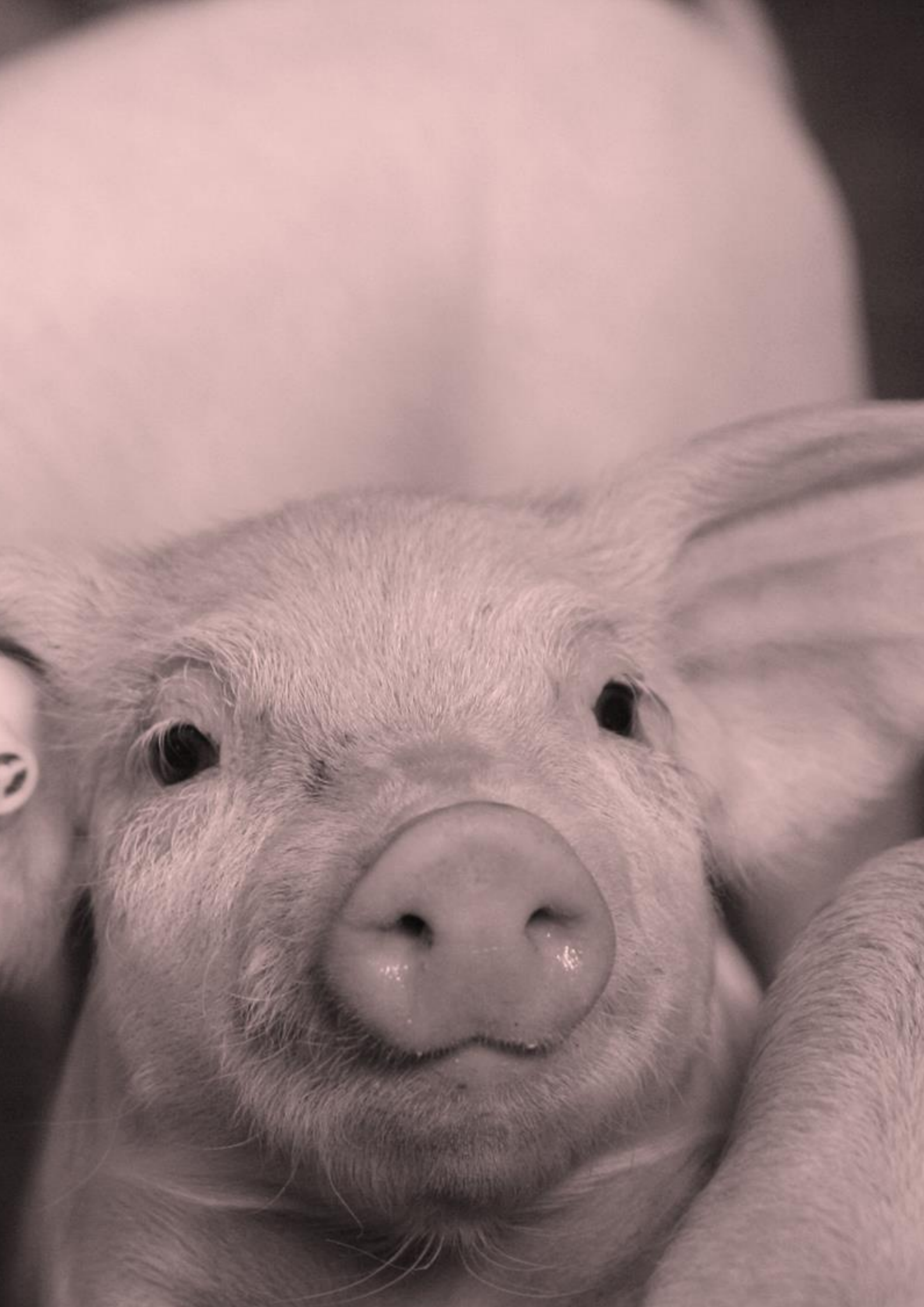

Supplement: Supplementary file 1 [file animals-12-01737-s001.zip › animals-1787600-SI.pdf]
